# Fyn Regulates Binding Partners of Cyclic-AMP Dependent Protein Kinase A

**DOI:** 10.3390/proteomes6040037

**Published:** 2018-09-29

**Authors:** Anna M. Schmoker, Samuel A. Barritt, Marion E. Weir, Jacqueline E. Mann, Tyler C. Hogan, Bryan A. Ballif, Paula B. Deming

**Affiliations:** 1 Department of Biology, University of Vermont, Burlington, VT 05405, USA; aschmoke@uvm.edu (A.M.S.); sbarritt@g.harvard.edu (S.A.B.); marion.weir@cellsignal.com (M.E.W.); 2 Department of Biomedical and Health Sciences, University of Vermont, Burlington, VT 05405, USA; jacmann@umich.edu (J.E.M.); Tyler.Hogan@uvm.edu (T.C.H.)

**Keywords:** protein kinase A, Fyn, AKAPs, LARP4, binding partners, mass spectrometry, SILAC, centrosome, Golgi apparatus

## Abstract

The cAMP-dependent protein kinase A (PKA) is a serine/threonine kinase involved in many fundamental cellular processes, including migration and proliferation. Recently, we found that the Src family kinase Fyn phosphorylates the catalytic subunit of PKA (PKA-C) at Y69, thereby increasing PKA kinase activity. We also showed that Fyn induced the phosphorylation of cellular proteins within the PKA preferred target motif. This led to the hypothesis that Fyn could affect proteins in complex with PKA. To test this, we employed a quantitative mass spectrometry approach to identify Fyn-dependent binding partners in complex with PKA-C. We found Fyn enhanced the binding of PKA-C to several cytoskeletal regulators that localize to the centrosome and Golgi apparatus. Three of these Fyn-induced PKA interactors, AKAP9, PDE4DIP, and CDK5RAP2, were validated biochemically and were shown to exist in complex with Fyn and PKA in a glioblastoma cell line. Intriguingly, the complexes formed between PKA-C and these known AKAPs were dependent upon Fyn catalytic activity and expression levels. In addition, we identified Fyn-regulated phosphorylation sites on proteins in complex with PKA-C. We also identified and biochemically validated a novel PKA-C interactor, LARP4, which complexed with PKA in the absence of Fyn. These results demonstrate the ability of Fyn to influence the docking of PKA to specific cellular scaffolds and suggest that Fyn may affect the downstream substrates targeted by PKA.

## 1. Introduction

The cAMP-dependent protein kinase A (PKA) is a serine/threonine kinase that plays many important roles in fundamental cellular processes, including cell proliferation, differentiation, and migration [1]. Many PKA interactors have evolved to sequester PKA within distinct subcellular locations for spatiotemporal regulation of its activity [2,3,4,5,6,7]. PKA is a heterotetrameric enzyme composed of two regulatory (R) and two catalytic (C) subunits, of which multiple isoforms exist: RI and RII, both of which have α and β isoforms, and Cα, Cβ, and Cγ. The C subunits remain inactive when bound to a pseudo-substrate consensus sequence within the R subunits, and are activated and canonically released upon the binding of cyclic adenosine monophosphate (cAMP).

The subcellular localization of PKA is mediated by A-kinase anchoring proteins (AKAPs), a class of scaffolding proteins that interacts with PKA-R subunits. The AKAP/PKA interaction interface is composed of the dimerization/docking (D/D) domain of the PKA-R subunit and the R-subunit binding domain of the AKAP [8]. Interestingly, the vast majority of AKAPs specifically bind the PKA-RII holoenzyme [9,10], although a select few are considered dual-affinity [11,12], binding both RI and RII subunits. AKAPs function to create discrete microenvironments of cAMP signaling [13].

Although PKA signaling has remained separate from that of Src family kinases (SFKs) in the canonical sense, recent work has uncovered crosstalk between PKA and SFKs [14,15,16,17]. It is well established that PKA is activated downstream of growth factors and activated receptor tyrosine kinases (RTKs). Activation of platelet-derived growth factor receptor (PDGFR) and epidermal growth factor receptor (EGFR) has been shown to regulate PKA activity by inducing phosphorylation of a conserved tyrosine, Y330 in human, of PKA-C [18]. Once activated, PKA, in certain systems, can phosphorylate and activate SFKs. PKA is known to phosphorylate Fyn on residue S21, which lies in a PKA consensus motif (RXXS/T). Phosphorylation of this site results in increased Fyn catalytic activity [19], and is required for cell migration and focal adhesion targeting of Fyn. Phosphorylation of the homologous site of Src (S21) drives ovarian cancer cell migration downstream of β-adrenergic receptors [20].

Recently, we have demonstrated a novel reciprocal interaction, in which Fyn and Src directly phosphorylate Y69 of the human α isoform of the catalytic PKA subunit (PKA–Cα), which results in increased PKA catalytic activity in vitro and in cells [21]. These findings led us to hypothesize that SFKs could modulate proteins in complex with PKA–Cα. Here, we employed a quantitative mass spectrometry approach to identify PKA–Cα binding partners whose binding was influenced by Fyn expression and catalytic activity.

## 2. Materials and Methods

### 2.1. Materials

Penicillin/streptomycin and Dulbecco’s modified eagle’s medium (DMEM) were obtained from Mediatech (Manassas, VA, USA). SILAC (stable isotope labeling of amino acids in cell culture) media was acquired from Thermo Scientific (Waltham, MA, USA), and fetal bovine serum (FBS), dialyzed FBS for SILAC experiments, and cosmic calf serum (CCS) were purchased from Hyclone (Logan, UT, USA). Unlabeled l-arginine (^12^C_6_, ^14^N_4_) and l-lysine (^12^C_6_, ^14^N_2_) were obtained from MP Biomedicals (Santa Ana, CA, USA), and heavy isotope-labeled l-arginine (^13^C_6_, ^15^N_4_) and l-lysine (^13^C_6_, ^15^N_2_) was purchased from Cambridge Isotope Laboratories, Inc. (Tewksbury, MA, USA). Packing material used for HPLC (high-performance liquid chromatography) was 5 μm C18-coated silica beads, 200 Å pore size, purchased from Michrom Bioresources, Inc. (Auburn, CA, USA). All additional reagents were purchased from Sigma (St. Louis, MO, USA), unless otherwise noted.

### 2.2. Plasmids and Antibodies

The *pcDNA_3.1_-PKACα-YFP* plasmid was a gift from Manuela Zaccolo from the University of Padua, previously described [22]. *pRK5* and *pRK5*-*Fyn* constructs [23] were acquired from Addgene (Cambridge, MA, USA): human *pRK5-Fyn WT* (wild type), *pRK5-Fyn KD* (K299M, kinase dead), and pRK5-Fyn *ΔSH3* (deletion of 76-141). The *pcDNA_3_-YFP* plasmid and *pcDNA_3.1_-EV* were obtained from Addgene (Cambridge, MA, USA). The *pFlag-CMV2 LARP4* and *pFLAG-CMV2* plasmids were a gift from Richard Maraia from the National Institute of Health, previously described [24]. The Fyn-Y3D mutant (Fyn-Y3D), possessing three tyrosine-to-aspartate mutations in the SH2 domain (Y185D, Y213D, and Y214D), was described previously [25].

The following primary antibodies were used for immunoblotting: α-Fyn (FYN3) and α-PKACα (C-20) were purchased from Santa Cruz Biotechnology (Dallas, TX, USA), α-phosphotyrosine (4G10) was purchased from EMD Millipore (Billerica, MA, USA), α-Myomegalin was obtained from Thermo Scientific (Waltham, MA, USA), α-AKAP9 and α-CDK5RAP2 were obtained from Bethyl Laboratories (Montgomery, TX, USA), and α-tubulin was purchased from Cell Signaling Technology (Danvers, MA, USA). Secondary antibodies (α-mouse-HRP and α-rabbit-HRP) were purchased from Jackson ImmunoResearch (West Grove, PA, USA).

### 2.3. Cell Culture

Human embryonic kidney (HEK) 293 cells were cultured in DMEM supplemented with 10% FBS, 50 U/mL penicillin, and 50 μg/mL streptomycin at 37 °C in 5% atmospheric CO_2_. U118-MG cells were cultured in RPMI 1640 medium supplemented with 10% FBS and 2 mM l-glutamine. For SILAC LC-MS/MS experiments, HEK 293 cells were cultured in labeled (heavy) or unlabeled (light) growth medium for one week prior to transfection to ensure complete protein labeling. SILAC media, lacking l-lysine and l-arginine were supplemented with 10% dialyzed FBS and antibiotics with 60 mg/L unlabeled l-proline, 100 mg/L of unlabeled or labeled (^13^C_6_, ^15^N_2_) l-lysine, and 100 mg/L of unlabeled or labeled (^13^C_6_, ^15^N_4_) l-arginine.

### 2.4. Transfection and Immunoprecipitation for Mass Spectrometry

HEK 293 cells were grown in either light or heavy DMEM as described above. Cells were transfected with 10 µg polyethylenimine (PEI) 24 h post-plating in 10-cm dishes with one of the following plasmid combinations (2.5 µg each) for control and experimental pairs: *YFP + EV* (heavy) and *YFP + Fyn WT* (light) as the control group, paired with *PKACα-YFP + EV* (heavy) and *PKACα-YFP + Fyn WT* (light) as the experimental group, or *YFP + Fyn KD* (heavy) and *YFP + Fyn WT* (light) as the control group, paired with *PKACα-YFP + Fyn KD* (heavy) and *PKACα-YFP + Fyn WT* (light). Cells were starved overnight in serum-free DMEM (heavy or light) 24 h post-transfection prior to lysis in 1 mL of NP40 lysis buffer (25 mM Tris pH 7.2, 137 mM NaCl, 25 mM NaF, 10 mM sodium pyrophosphate, 10% glycerol, 1% IGEPAL), with 1 mM dithiothreitol, 1 mM Na_3_VO_4_, 1X HALT Protease inhibitor cocktail (Thermo Scientific, Waltham, MA, USA), and 1 mM β-glycerophosphate. Protein concentrations were determined using a Pierce BCA Protein Assay Kit (Thermo Scientific, Waltham, MA, USA). A total of 8 mg protein extract per treatment group was first pre-cleared with glutathione sepharose beads (G-Biosciences, St. Louis, MO, USA), rocking for 30 min at 4 °C. Clarified extracts were immunoprecipitated with 5 µg α-GFP (Life Technologies, Carlsbad, CA, USA) pre-bound to magnetic Protein G Dynabeads (Thermo Scientific, Waltham, MA, USA), while rocking for 4 h at 4 °C. Immune complexes were subjected to five washes with 1 mL NP40 lysis buffer, and bound proteins were eluted and denatured at 95 °C for 5 min in Laemmli sample buffer.

### 2.5. Peptide Processing for Mass Spectrometry

Light and heavy conditions of experimental pairs outlined above were combined following immunoprecipitation and eluted proteins were run on a 10% SDS-PAGE gel. Gels were stained with Coomassie Brilliant Blue and each lane was divided into 14 regions by molecular weight for analysis via liquid chromatography tandem mass spectrometry (LC-MS/MS). Preparation of peptides for LC-MS/MS was described previously [26]. Briefly, excised bands were de-stained prior to an in-gel digestion with sequencing grade, modified trypsin (Promega, Madison, WI, USA). Tryptic peptides were extracted and separated on a reverse-phase HPLC column (length = 12 cm × 100 µm) packed in-house with 5 µm C18 beads (pore size = 200 Å) (Michrom Bioresources, Inc., Auburn, CA, USA) prior to analysis via a linear ion trap-orbitrap (LTQ-Orbitrap Discovery; resolution = 3 × 10^4^, scan speed = 1 Hz) mass spectrometer fitted with a Finnigan Surveyor Pump Plus and Micro AS autosampler (Thermo Electron, San Jose, CA, USA) and controlled with Xcalibur™ 2.1 Software (Thermo Fisher Scientific, Inc., Waltham, MA, USA). The precursor scan (365–1600 *m/z*), acquired in the orbitrap mass analyzer, was followed by ten low energy collision-induced dissociation (CID) tandem mass spectra in the linear ion trap. All spectra were obtained in centroid, with dynamic exclusion parameters as follows: repeat count = 2, repeat duration = 30 s, exclusion list size = 100, exclusion duration = 120 s, exclusion width = ±1.5 *m/z*.

SEQUEST searches were performed using a forward and reverse 2011 Uniprot Human Protein database requiring tryptic peptides and permitting the modification of serine, threonine and tyrosine (+79.966 Da), methionine (+15.995 Da), cysteine (+71.037 Da), arginine (+10.008 Da) and lysine (+8.014 Da) with a mass tolerance of ±4 ppm and unique ΔCorr of ≥0.2. Proteins were considered quantifiable if identified by three or more peptides with a signal-to-noise ratio of ≥5 for either the heavy or light peptide. Peptides identified in the YFP control lanes were removed from the experimental dataset unless they were present at an abundance five times greater in the experimental set than in the YFP control. A complete list of quantified proteins can be found in Tables S1 and S2. Phosphopeptides were removed prior to protein quantification and analyzed separately (Table S3). Heavy-to-light ratios (H:L) of monoisotopic peak intensities of precursor ions were quantified using Vista [27]. Average peptide ratios per protein were normalized to that of the loading control (YFP), and the average H:L of each protein was compared to YFP using a two-tailed paired student’s *t*-test with a Benjamini–Hochberg (B–H) correction to give the B–H critical value. Proteins with a B–H critical value < 0.05 and 2-fold or more change in H:L were considered moderately increased or decreased in binding. Normalized ratios were Log_2_-transformed post-analysis for visualization.

Protein functions were assigned based on annotations in PhosphoSitePlus [28] and UniProt [29]. Known PKA or Fyn binding partners were identified by the Biogrid [30] curations as well as primary literature searches. Substrates of PKA and Fyn were found using the “Substrates of” tool on PhosphoSitePlus [28].

### 2.6. Transfection and Immunoprecipitation for Western Blotting

HEK 293 cells were grown in normal DMEM and transfected with PEI as described in Section 2.4, or via calcium phosphate precipitation, but with 5 µg of each plasmid. An immunoprecipitation was performed as described in Section 2.4, with the exception that lysates (1–1.5 mg) were either incubated with α-GFP or α-PKA–Cα overnight and then with Protein G Dynabeads for 2 h. Immunoprecipitates were separated via SDS-PAGE prior to Western blotting. Proteins were transferred to a nitrocellulose membrane with a Trans-Blot Turbo RTA Transfer Kit (Bio-Rad Laboratories, Hercules, CA, USA), and membranes were subsequently blocked in 1% BSA/1X Tris-buffered saline + 0.1% Tween-20 solution (TBST), rocking for one hour at 25 °C. Primary antibodies were diluted in TBST with 1% BSA and incubated overnight at 4 °C while rocking. Membranes were washed five times for five minutes each in TBST. Secondary antibodies were diluted to 1:5000 (α-mouse-HRP) or 1:10,000 (α-rabbit-HRP) in TBST, and incubated while rocking for one hour at 25 °C. Following five washes in TBST, membranes were briefly incubated in SuperSignal West Pico Chemiluminescent Substrate (Thermo Scientific, Waltham, MA, USA) and imaged with a PXi4 EZ imaging system (Syngene, Frederick, MD, USA) or exposed to X-ray film. Similarly, whole cell extracts (WCEs) were denatured as described above and analyzed via SDS-PAGE and Western blotting.

## 3. Results

### 3.1. Identification of Fyn-Mediated PKA–Cα Binding Partners

**Figure 1:**
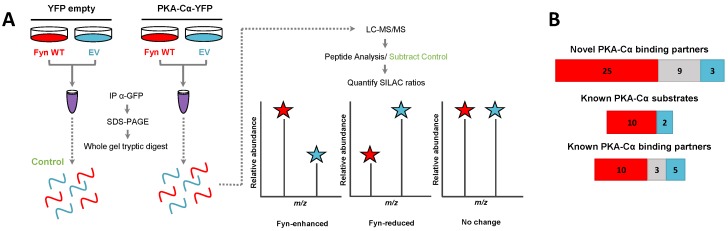
SILAC experimental design for the characterization of PKA–Cα binding partners in the presence/absence of Fyn. (**A**) SILAC experimental workflow. Conditions of control (YFP alone) and experimental (PKA–Cα-YFP) SILAC pairs are given. Light SILAC culture conditions (with Fyn WT) are indicated in red, while heavy conditions (without Fyn WT) are shown in blue. After immunoprecipitation (IP; α-GFP) SILAC pairs were combined and bound proteins were separated via SDS-PAGE prior to a tryptic digest. Peptides were analyzed via LC-MS/MS and SILAC heavy-to-light (H:L) ratios were calculated. Proteins also identified in the control condition (YFP alone) were removed from the experimental (PKA–Cα-YFP) set. Expected outcomes are as follows: PKA-C interactors are either Fyn-enhanced binding partners with significantly increased binding in the presence of Fyn (H:L ratio < 1), Fyn-reduced binding partners with significantly decreased binding in the presence of Fyn (H:L ratio > 1), or exhibit no statistically significant difference between the two conditions (H:L ratio = 1); (**B**) experimentally identified PKA–Cα interactors and the effect of Fyn on known and novel PKA–Cα binding partners and known PKA–Cα substrates. Red indicates Fyn-enhanced binding, blue indicates Fyn-reduced binding, and gray indicates no significant change in binding.

We recently identified a novel interaction between the tyrosine kinase Fyn and PKA, in which Fyn co-immunoprecipitates with and phosphorylates Y69 of the α isoform of the catalytic PKA subunit (PKA–Cα) [21]. Furthermore, expression of Fyn in cells led to increased phosphorylation of proteins at motifs preferentially phosphorylated by PKA [21]. These results led us to hypothesize that Fyn could modulate the binding of PKA–Cα interactors, and conceivably increase their phosphorylation by PKA. To determine whether the co-expression of Fyn would alter proteins in complex with PKA–Cα, we employed a quantitative mass spectrometry-based approach to identify proteins that bound to PKA–Cα differentially in the presence of Fyn. Briefly, HEK 293 cells grown in heavy or light SILAC media were co-transfected with mammalian expression vectors for *PKACα-YFP* and *WT Fyn* (light) or *PKACα-YFP* and *empty vector* (heavy) (Figure 1A). Following α-GFP immunoprecipitation of extracts for the two states independently, immune complexes were combined and subjected to SDS-PAGE. Note that the α-GFP antibody also recognizes YFP. Proteins from the entire lane of the gel were digested with trypsin prior to analysis via LC-MS/MS. Heavy-to-light ratios (H:L) of peptides were averaged across each binding partner and normalized to the H:L of PKA–Cα-YFP. Proteins in α-GFP immune complexes from control conditions (Figure 1A) were subtracted from the list of potential PKA–Cα binding partners unless they had five times the number of peptide spectral matches in the experimental condition over the control.

**Figure 2:**
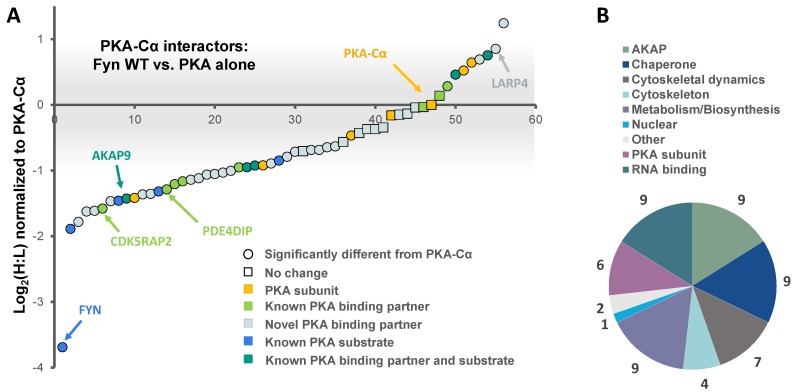
The effect of the tyrosine kinase Fyn on proteins in complex with PKA–Cα. (**A**) SILAC (H:L) ratios of proteins in complex with PKA–Cα were normalized to the H:L of PKA–Cα. H:L ratios were log_2_-transformed, and plotted in rank order. Data points denote single proteins, and data point shape indicates whether the H:L ratio of a given protein exhibited a statistically significant difference from (circular) or was the same as (square) that of PKA–Cα. Known and novel PKA interactors and known PKA substrates are highlighted by the colors indicated in the key. The gray bar indicates the range of Log_2_ values that correspond to less than a 2-fold change (increase or decrease) from the H:L ratio of PKA–Cα; (**B**) representation of the various cellular functions for the PKA–Cα interactors identified.

In addition to PKA–Cα, a total of 55 PKA–Cα interactors were identified, of which 35 were found significantly increased in the presence of Fyn, eight were reduced by Fyn, and 12 showed Fyn-independent binding to PKA–Cα (Figure 1B and Figure 2A, Table S1). Proteins were considered enriched in a given environment if the absolute value of the Log_2_-transformed H:L ratio was greater than one, corresponding to a fold change greater than two (Figure 2A). Interestingly, the majority of the identified PKA–Cα binding partners were Fyn-enhanced. This included several known PKA–Cα interactors including the AKAPs myomegalin (PDEDIP4), AKAP9, and CDK5RAP2 (Figure 2A, Table S1). Notably, Fyn co-immunoprecipitated with PKA–Cα when co-expressed, demonstrating the physical interaction between Fyn and PKA (Figure 2A) [21].

Functional classification of all interacting partners revealed strong representation of AKAPs, PKA subunits, and proteins involved in cytoskeletal dynamics, in addition to other biological processes (Figure 2B). Of the 56 proteins identified, 37 were novel PKA–Cα interactors (Figure 1B). While the majority of the interactors showed increases in PKA-C complexes with co-expression of Fyn, three novel interactors were significantly decreased when Fyn was co-expressed with PKA–Cα (Figure 2A, Table S1).

### 3.2. Effect of Fyn-Activity on Proteins in Complex with PKA–Cα

**Figure 3:**
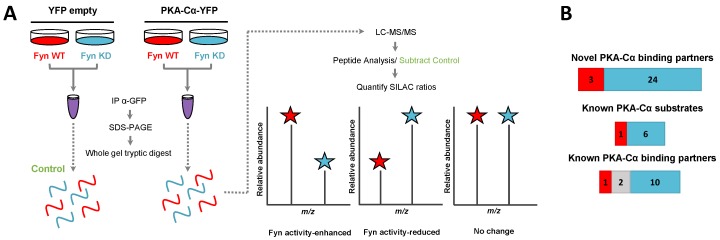
SILAC experimental design for the characterization of the effect of Fyn activity on PKA–Cα binding partners. (**A**) SILAC experimental workflow. Conditions of control (YFP alone) and experimental (PKA–Cα-YFP) SILAC pairs are indicated. Light SILAC culture conditions (with Fyn WT) are shown in red, while heavy conditions (with Fyn KD) are represented in blue. The workflow is described in detail in the legend to Figure 1. Expected outcomes are as follows: PKA-C interactors either exhibit enhanced binding in the presence of Fyn activity (H:L ratio < 1), reduced binding in the presence of Fyn activity (H:L ratio > 1), or exhibit no statistically significant difference between the two conditions (H:L ratio = 1); (**B**) experimentally identified PKA-C interactors and the effect of Fyn on known and novel PKA–Cα binding partners and known PKA–Cα substrates. Red indicates Fyn activity-enhanced binding, blue indicates Fyn activity-reduced binding, and gray indicates no significant change in binding.

We then asked whether any of these Fyn-mediated interactors were dependent upon Fyn catalytic activity. We conducted a second SILAC experiment to directly compare the influence of Fyn tyrosine kinase activity on PKA–Cα binding partners to that of a kinase dead (KD) mutant (K299M). HEK 293 cells grown in heavy or light SILAC media were co-transfected with expression plasmids for *PKACα-YFP* and *Fyn WT* (light) or *PKACα-YFP* and *Fyn KD* (heavy) (Figure 3A). Proteins in the PKA–Cα-YFP immune complexes were analyzed as described above.

**Figure 4:**
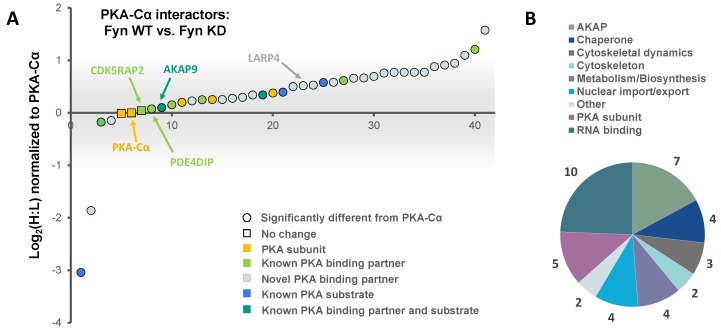
The effect of Fyn catalytic activity on proteins in complex with PKA–Cα. (**A**) SILAC heavy-to-light (H:L) ratios of proteins in complex with PKA–Cα were normalized to that of PKA–Cα, log_2_-transformed, and plotted in rank order. The significance of data point shape and color are outlined in the key. The gray bar indicates the range of Log_2_ values that correspond to less than a 2-fold change (increase or decrease) from the H:L ratio of PKA–Cα; (**B**) biological functions represented across the PKA–Cα interactors identified.

In addition to PKA–Cα, a total of 40 PKA–Cα interacting partners were identified, of which four were enhanced in binding in the presence of active Fyn, 34 were reduced, and two showed no significant change (Figure 3B, Table S2). Surprisingly, only two of these proteins were appreciably enriched in the co-expression of PKA–Cα and Fyn WT (Figure 4A), suggesting that phosphorylation events downstream of Fyn activity reduce the binding of the majority of the identified PKA–Cα interactors. Even so, proteins that exhibited increased binding to PKA–Cα in the presence of Fyn KD were not highly enriched, possessing low, though positive, Log_2_ ratios (Figure 4A). Among those with no appreciable change in binding in Fyn active/inactive conditions were Fyn-enhanced PKA–Cα interactors identified in the previous experiment: CDK5RAP2, PDE4DIP, and AKAP9. Although Fyn was not identified by LC-MS/MS in either the heavy or light condition in the second experiment, Fyn expression was confirmed via Western blotting (Figure S1). As relatively few Fyn peptides were identified in the first SILAC experiment (Table S1), it is not necessarily surprising that Fyn was not observed via LC-MS/MS in the second experiment.

Functional classification revealed a similar range of cellular roles of PKA–Cα binding partners as that observed in the previous experiment, with strong representation of PKA subunits and AKAPs (Figure 4B). In total, 27 of the identified proteins in complex with PKA–Cα were novel binding partners (Figure 3B). While the majority of these novel PKA–Cα interactors were reduced in binding when PKA–Cα was co-expressed with WT Fyn as compared to KD Fyn, three (ANXA1, KRT77, LARP1B) exhibited significantly increased binding (Figure 4A, Table S2). 

**Figure 5:**
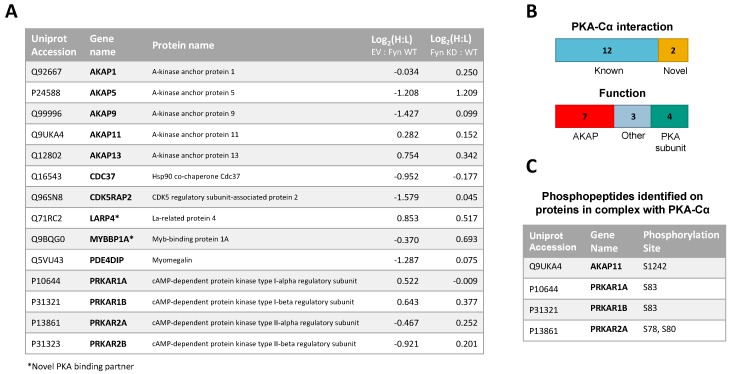
(**A**) PKA–Cα interactors common among LC-MS/MS experiments conducted in this study. H:L ratios of proteins are given for each experiment; (**B**) of the 14 common proteins, two were novel PKA–Cα interactors, four were PKA subunits and seven were AKAPs; (**C**) phosphopeptides identified on proteins in complex with PKA–Cα. All phosphorylation sites were enriched in Fyn-active conditions. A table of phosphopeptides with respective H:L ratios and spectral counts (Table S3) and the corresponding annotated fragmentation spectra (Figure S2) are included in the supplementary material.

A total of 14 PKA–Cα interactors were common across both SILAC experiments conducted (Figure 5A). Among these were four expected PKA subunits, seven AKAPs, one additional known PKA–Cα binding partner, and two novel binding partners (Figure 5A,B). Several of these proteins, including AKAP5, AKAP9, PDE4DIP, and CDKRAP2, exhibited strong Fyn-mediated binding in the first experiment, all of which except AKAP5 did not appear to be affected by Fyn activity, as observed from the second experiment (Figure 5A). This suggests the interaction of AKAP9, PDE4DIP, and CDKRAP2 with PKA–Cα may be enhanced by the Fyn/PKA–Cα physical interaction (Figure 2A). Interestingly, AKAP5 bound more strongly to PKA–Cα in the presence of Fyn KD, suggesting that Fyn-induced phosphorylation of PKA–Cα, AKAP5, or associated proteins may lead to some degree of dissociation of AKAP5 from the PKA–Cα complex. To a lesser extent, PKA-RIIα and β subunits exhibited a modest reduction in binding in the presence of Fyn, although this also was independent of Fyn activity.

In addition to identifying Fyn-enhanced/reduced interactors of PKA–Cα, we observed changes in serine/threonine phosphorylation of specific sites on four proteins (one AKAP and three PKA-R subunits) in complex with PKA–Cα (Figure 5C). In the experiment comparing binding partners of PKA–Cα with (light) or without (heavy) WT Fyn, H:L ratios of peptides housing pS1242 of AKAP11 and pS83 of PKA-RIα were significantly lower (Fyn-enhanced) than the average H:L ratios of the respective full proteins (Table S3, annotated spectra in Figure S2A,B). Similarly, H:L ratios of peptides housing PKA-RIα, PKA-RIβ, and PKA-RIIα phosphorylation sites were significantly lower than that of each full protein in the experiment comparing PKA–Cα interactors with Fyn WT (light) and Fyn KD (heavy) (Table S3, annotated spectra in Figure S2C–F). Perspectives on the implications of these sites of differential phosphorylation are included in the discussion (Section 4).

### 3.3. Biochemcial Validation of Fyn-Dependent Binding Partners

We chose three known PKA–Cα binding partners with enhanced binding in the presence of Fyn for validation in a co-immunoprecipitation experiment. Three of the seven AKAPs that were identified in both experiments were of particular interest, as they are known to co-localize and function at the Golgi apparatus and centrosome. AKAP9, Phosphodiesterase 4D interacting protein (PDE4DIP, also myomegalin) and CDK5 Regulatory Subunit Associated Protein 2 (CDK5RAP2) participate in microtubule organization, facilitating the nucleation of microtubules through assembly of γ-tubulin ring complexes (γ-TURCs) [31]. These three known PKA–Cα interactors demonstrated strongly increased binding to PKA–Cα in the presence of Fyn, but this was independent of Fyn activity (Figure 5A). In follow-up experiments, we investigated their interaction with PKA-C biochemically.

**Figure 6:**
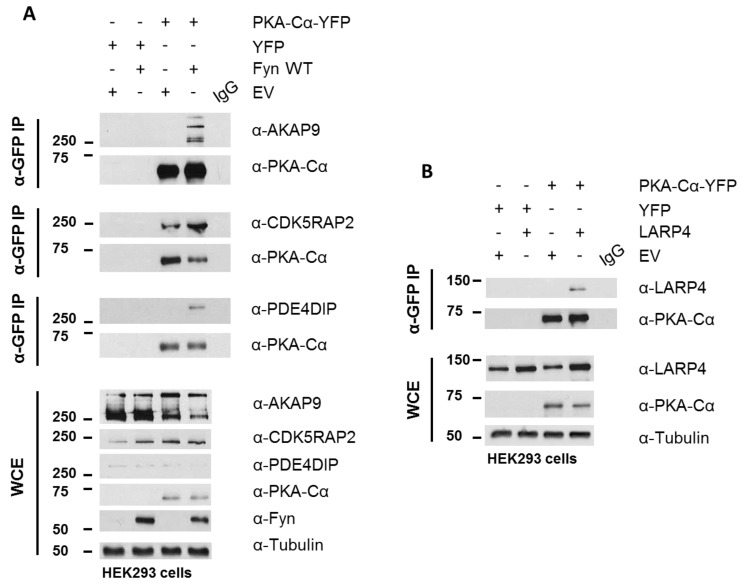
Biochemical validation of PKA–Cα interacting partners. (**A**) AKAP9, CDK5RAP2, and PDE4DIP interact with PKA–Cα in a Fyn-dependent manner. HEK 293 cells were transfected with the indicated plasmids and cell lysates were analyzed via SDS-PAGE and immunoblotting, either for expression levels in the whole cell extract (WCE) or for co-immunoprecipitation (IP; α-GFP) with PKA–Cα. Western blotting of WCEs display levels of endogenous AKAP9, CDK5RAP2, PDE4DIP and expressed PKACα-YFP, and Fyn. The PKA–Cα-YFP IP (α-GFP) from whole lysates reveal co-IP of AKAP9, CDK5RAP2, and PDE4DIP in the presence of Fyn; (**B**) LARP4 co-immunoprecipitates with PKA–Cα. The indicated plasmids were expressed in HEK 293 cells prior to analysis via SDS-PAGE and immunoblotting, either for expression (WCE) or for co-IP (α-GFP) with PKA–Cα-YFP. Western blotting of PKA–Cα-YFP IP (α-GFP) from whole lysates reveal co-IP of LARP4. An IgG control was performed on mixed lysates from each sample in both (**A**) and (**B**).

PKA–Cα-YFP was expressed in HEK 293 cells cultured in normal growth medium with and without Fyn WT. Whole cell extracts (WCEs) were assessed for expression levels of endogenous AKAPs following SDS-PAGE and Western blotting (Figure 6A). Immunoblotting of PKA–Cα-YFP immunoprecipitates (IP α-GFP) for AKAP9, PDE4DIP, and CDK5RAP2 confirmed increased binding of all three AKAPs when Fyn was co-expressed with PKA–Cα (Figure 6A). In combination with the Log_2_-transformed H:L ratios observed in the Fyn WT/KD experiment (Figure 4A), these data suggest that Fyn enhances the association of PKA–Cα with the macromolecular AKAP complex through physical complexation in a manner that is independent of Fyn kinase activity.

Although AKAP9, PDE4DIP, and CDK5RAP2 are known interactors of the PKA holoenzyme, we identified two novel PKA–Cα interactors common to both SILAC experiments, LARP4 and MYBBP1A (Figure 4A), as well as a number of additional novel PKA binding partners that did not overlap between the two experiments (Tables S1 and S2). LARP4 is known to stabilize mRNA through binding to their poly-A tract [24] but has also been identified as a negative regulator of cell migration and invasion in a prostate cancer cell line [32]. Interestingly, LARP4 exhibited moderately reduced binding to PKA in the presence of Fyn WT, and this appeared to be affected by Fyn activity (Figure 5A). Therefore, we chose to test its ability to co-immunoprecipitate with PKA–Cα in a co-expression experiment. LARP4 and PKA–Cα-YFP were co-expressed in HEK 293 cells (Figure 6B) and α-GFP immunoprecipitates were subjected to SDS-PAGE and Western blotting. Indeed, co-expressed LARP4 was identified in the PKA–Cα immune complex (Figure 6B).

**Figure 7:**
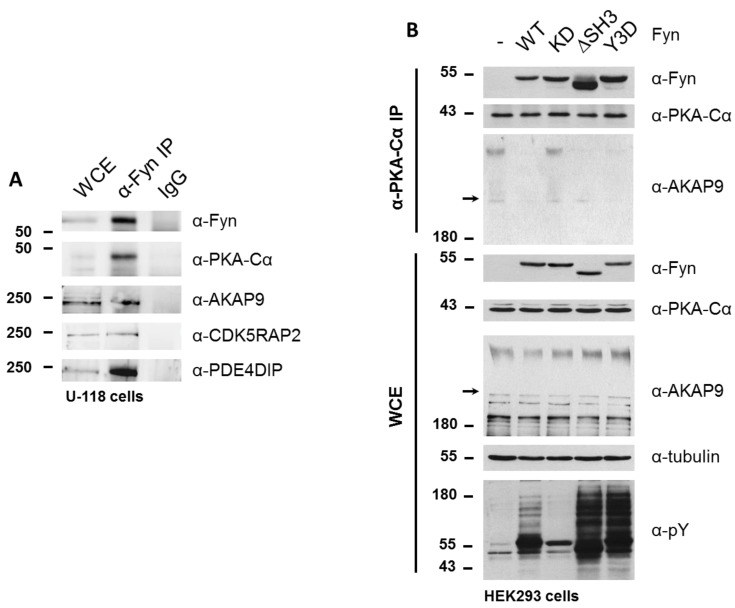
Fyn participates in PKA/AKAP complexes. (**A**) Endogenous PKA and AKAPs co-immunoprecipitate with Fyn in a glioblastoma cell line. Endogenous Fyn was immunoprecipitated from glioblastoma cells (U-118), which express high levels of Fyn, and analyzed via SDS-PAGE and immunoblotting. Whole cell extracts, α-Fyn immunoprecipitations, and IgG controls were run on the same gel. PKA–Cα, AKAP9, CDK5RAP2, and PDE4DIP were all detected in the immune complex; (**B**) Fyn activity mediates release of endogenous PKA–Cα from the AKAP9-anchored complex. WT and mutant alleles of Fyn (KD, ΔSH3, Y3D) were expressed in HEK cells, and extracts were analyzed via SDS-PAGE and immunoblotting before (WCE) or after immunoprecipitation of endogenous PKA–Cα (α-PKA–Cα). Both the stacking and separating layers were transferred to a nitrocellulose membrane for AKAP9 immunoblotting. Arrows in the AKAP9 blots of the IP and WCE denote the interface between stacking and separating layers.

To determine whether Fyn participates in the PKA/AKAP complex, we immunoprecipitated endogenous Fyn from a glioblastoma cell line (U-118), which expresses high levels of Fyn. In addition to endogenous PKA–Cα, we detected the co-immunoprecipitation of endogenous AKAP9, CDK5RAP2, and PDE4DIP in this cell line (Figure 7A).

Given that the Fyn-enhanced association of PKA with AKAP9, CDK5RAP2, and PDE4DIP was observed to be independent of Fyn kinase activity in experiments were Fyn and PKA–Cα were co-expressed, in combination with the detection of endogenous Fyn in complex with PKA and AKAPs, we hypothesized that Fyn could serve as an adaptor protein to facilitate the formation of the PKA holoenzyme/AKAP complex. To test this hypothesis, we expressed WT and mutant Fyn alleles in HEK cells to determine the ability of these mutants to bind endogenous PKA–Cα, as well as their effect on the anchorage of PKA–Cα to the AKAP/holoenzyme complex. In addition to the wild-type (Fyn WT) and kinase-dead (Fyn KD) alleles, we explored the interaction of endogenous PKA–Cα with a construct of Fyn missing the SH3 domain (Fyn ΔSH3) as well as one with three tyrosine-to-aspartate mutations in the SH2 domain of Fyn (Fyn Y3D), which prevents SH2-mediated binding to phosphotyrosine docking sites [25]. The products of all four Fyn alleles co-immunoprecipitated with endogenous PKA–Cα, with Fyn ΔSH3 exhibiting enhanced binding to PKA–Cα relative to the products of the other alleles (Figure 7B). Given that both Fyn ΔSH3 and Fyn Y3D showed comparable increases in phosphorylation of endogenous substrates (Figure 7B), and given wild type and kinase-dead variants of Fyn showed similar binding, the enhanced binding of Fyn ΔSH3 to PKA cannot be explained simply by a correlation to catalytic activity. Instead, these data suggest that the Fyn SH3 domain might sterically hinder Fyn from interacting with PKA.

We were surprised, however, to observe a Fyn activity-dependent interaction between endogenous PKA–Cα and AKAP9. High molecular weight isoforms of AKAP9 (approximately 350 kDa and 450 kDa) were detected in the immune complex (α-PKAC-α) at similar levels when Fyn KD was expressed and in the absence of Fyn over-expression (Figure 7B). However, when expressing Fyn WT and the mutant alleles Fyn ΔSH3 and Fyn Y3D, which exhibit increased catalytic activity over Fyn WT (Figure 7B, α-pY blot), AKAP9 levels were reduced in the PKA–Cα complex (Figure 7B). Although these findings at first appeared to be in conflict with the results obtained from the quantitative mass spectrometry results and co-immunoprecipitations shown in Figure 4, Figure 6 and Figure 7, the apparent discrepancies could be due to the differences in cellular Fyn and PKA–Cα activities and expression levels (see Discussion below).

## 4. Discussion

We have recently identified an interaction between two protein kinases, PKA and the SFK Fyn, in which Fyn phosphorylates the catalytic subunit of the PKA holoenzyme at Y69 [21]. These observations, in combination with the mutual PKA-induced phosphorylation of Fyn S21 [19], presented the possibility for a regulatory relationship between these protein kinases. Here, we further investigated the consequences of the Fyn/PKA interaction using a quantitative LC-MS/MS approach. We identified both known and novel interactors of the α isoform of the catalytic PKA subunit, some of which are induced to associate or dissociate with PKA–Cα in the presence of Fyn catalytic activity or by simple expression of Fyn through a Fyn–PKA physical interaction. We observed enhanced PKA–Cα binding of 35 of 55 total proteins in the presence of Fyn (Figure 1B and Figure 2A). Contrary to our hypothesis that this event was mediated by Fyn kinase activity alone, the majority of interactors that were found to co-immunoprecipitate with PKA–Cα were not enhanced by Fyn activity (Figure 3B and Figure 4A) when PKA–Cα and Fyn were over-expressed. The identification of Fyn in complex with PKA–Cα (Figure 2A) suggests that Fyn could function as an adaptor protein, facilitating the formation of a large multimeric complex comprised of PKA–Cα and various interacting partners.

Several known PKA substrates, including Fyn and AKAP9, were enriched in the condition in which Fyn was co-expressed with PKA–Cα (Figure 1B and Figure 2C). Intriguingly, two proteins exhibited decreased binding in the presence of Fyn, namely, AKAP13 and MAP2 (Table S1). The Rho–GEF activity of AKAP13 (also known as AKAP-Lbc) is inhibited by phosphorylation of S1565 by cAMP-activated PKA [33]. The observed decrease in binding of AKAP13 to PKA–Cα driven by Fyn expression could result from the dissociation of the C subunit after Fyn-induced PKA activation. In the case of MAP2, PKA-induced phosphorylation of MAP2 is known to disrupt the microtubule-MAP2 interaction and promote MAP2 localization to actin fibers in the cell periphery [34]. Interestingly, MAP2 is known to bind both PKA and Fyn [35,36], and PKA-induced phosphorylation of Fyn S21 targets Fyn to focal adhesions [19]. The finding that the MAP2/PKA interaction was decreased in the presence of Fyn, despite the detection of Fyn peptides in complex with PKA, suggests that PKA activity could promote dissociation of MAP2 from the PKA holoenzyme and microtubules. Given the known interaction between Fyn and MAP2, MAP2 dissociation from the PKA holoenzyme could promote the formation of a complex between MAP2 and a subset of Fyn molecules that localizes to the cell periphery.

The three Fyn-enhanced proteins selected for biochemical characterization are known AKAPs of PKA. Although AKAP9, PDE4DIP, and CDK5RAP2 are known to anchor the PKA holoenzyme at the Golgi apparatus and centrosome to facilitate microtubule organization [31], this work characterizes the interaction of these proteins with PKA as Fyn-mediated, and demonstrates that Fyn exists in complex with PKA and these AKAPs in a glioblastoma line (Figure 7A). Several reports have demonstrated various interactions between these three AKAPs. PDE4DIP, or myomegalin, is a dual-specificity AKAP [37] known to co-localize with AKAP9 (also AKAP350, AKAP450, Yoatio) and PKA at the centrosome [38,39,40]. Myomegalin has been shown to be required for the localization of AKAP9 to the Golgi [38]. CDK5RAP2 (also CEP215) is one of 32 members of a family of proteins important in cell cycle progression through the generation of mitotic spindle poles [41]. CDK5RAP2 is targeted to the Golgi apparatus and centrosome through its C-terminus, and helps to maintain centrioles in close proximity prior to separation in a G2/M phase [41]. CDK5RAP2 also contains a CNN1 domain that functions in the recruitment of AKAP9 to the centrosome, allowing for proper anchorage of mitotic spindle proteins. Interestingly, the SFK Src is necessary for microtubule nucleation and regrowth in human fibroblasts [42]. Together with the data presented here, these studies suggest that SFKs could play important roles in the localization and function of the AKAP/PKA complex in microtubule dynamics.

**Figure 8:**
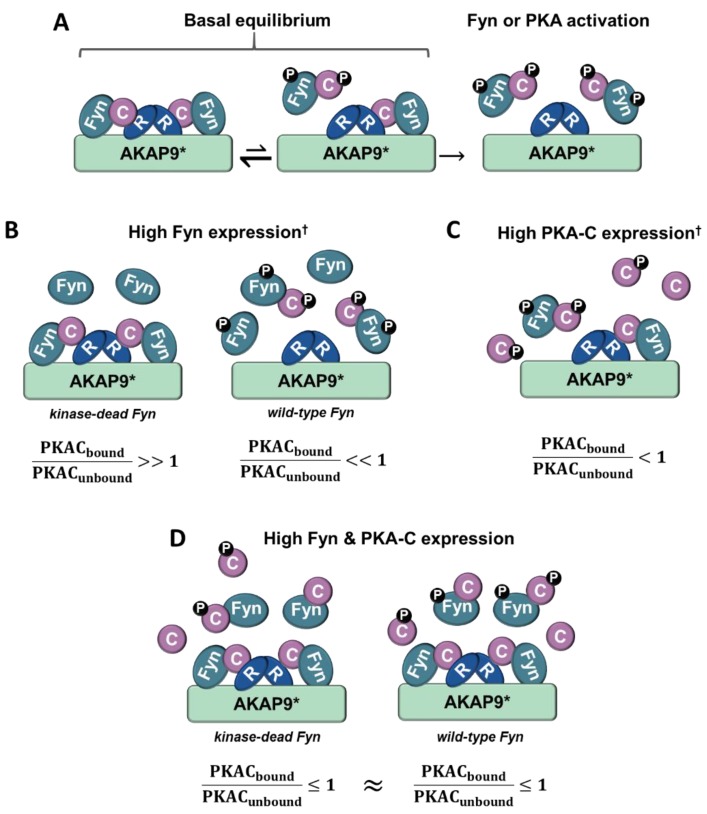
Model for Fyn and PKA-C concentration-dependent anchorage to the holoenzyme/AKAP complex. (**A**) In cells expressing moderate levels of Fyn and PKA-C, the majority of PKA-C/Fyn complexes are anchored to AKAPs. Upon PKA or Fyn activation, PKA-C/Fyn is released from the AKAP complex. (**B**) In cells expressing high levels of Fyn that lacks kinase activity and moderate PKA-C levels, the majority of PKA-C subunits remain bound to AKAP complexes. However, high levels of active Fyn can lead to Fyn autophosphorylation and activation, and subsequent activation of PKA-C and release of PKA-C/Fyn. (**C**) In cells expressing high levels of PKA-C, catalytic subunits are similarly activated, leading to the release of PKA-C/Fyn from AKAP complexes. (**D**) In cells expressing high levels of PKA-C and Fyn, a pool of PKA-C/Fyn remain bound to AKAPs despite the unregulated activation of Fyn and PKA due to concentration-dependent equilibria. PKA-C = C, PKA-R = R, † = high levels of Fyn or PKA-C can induce unregulated activity, * = this model is corroborated experimentally with AKAP9, although it could be representative of PKA/Fyn regulation of other AKAPs.

Although the PKA/AKAP interactions were observed to be independent of Fyn activity in conditions where PKA–Cα and Fyn were highly expressed (Figure 2A, Figure 4A and Figure 6A), we were surprised to observe strong Fyn activity-mediated association of endogenous PKA–Cα with AKAP9 (Figure 7B). We hypothesize that this effect is the result of differential PKA–Cα and Fyn activity and expression levels across the experiments conducted in this study. Our observations could hold important biological relevance when levels of Fyn and PKA-C vary across cell types, particularly as both kinases can exhibit unregulated activity when expressed at high levels. This leads us to develop the model outlined in Figure 8. In cells expressing moderate levels of Fyn and PKA–Cα, the basal equilibrium of Fyn/PKA–Cα anchorage to R subunits and AKAPs would favor the AKAP-bound fraction (Figure 7A,B), given the substantial molar excess of R subunits relative to C subunits [43]. Upon either PKA or Fyn activation, the C subunits and Fyn would dissociate from the AKAP complex (Figure 8A). In cells with high levels of Fyn, this equilibrium would favor free Fyn/PKA–Cα, unless Fyn was unable to auto-activate (Figure 8B), as was observed in the expression of Fyn KD alone (Figure 7B). In cells expressing high PKA–Cα, as in experiments with PKA–Cα-YFP expression alone (Figure 2A and Figure 6A), unregulated PKA activity would lead to C subunit dissociation from AKAP complexes (Figure 8C). Finally, in cells expressing high levels of Fyn and PKA–Cα, as in experiments with PKA–Cα-YFP and Fyn co-expressed (Figure 2A and Figure 6A), a subset of PKA–Cα/Fyn would remain bound to the AKAP complex, despite unregulated kinase activity. These findings underscore the importance of querying interactions of endogenous proteins in addition to those observed in over-expression studies, although a combination of these approaches would provide context for complexes that could form in cells expressing different levels of endogenous PKA–Cα or Fyn, or when either is aberrantly expressed in a disease state.

Several cytoskeletal and centrosomal proteins that would function in this macromolecular complex, including tubulin subunits, were identified in both SILAC experiments (Tables S1 and S2). CEP170 was among the most highly Fyn-enhanced PKA binding partners (Table S1). CEP170 localizes to centrosomes and microtubules and is involved in cytoskeletal organization [44]. The binding of Microtubule Associated Protein RP/EB Family Member 1 (MAPRE1; also EB1), which regulates polymerization at the growing end of microtubules [45], was also highly regulated by Fyn. Interestingly, MAPRE1 (EB1) was recently found to exist in a large multimeric complex with a short isoform of myomegalin, AKAP9, and CDK5RAP2 [46]. Other highly Fyn-enhanced PKA–Cα interactors included the cytoskeletal proteins vimentin (VIM) and emerin (EMD), which both associate with the centrosome [47,48] (Table S1). The effect of Fyn activity on the association of these proteins with PKA–Cα further supports our hypothesis that Fyn may act to preferentially localize PKA to centrosomal or Golgi-localized regulatory complexes.

In addition to characterizing Fyn-induced changes in the association of known PKA interactors, we identified a novel moderately Fyn-reduced PKA–Cα interactor, LARP4. Although the specific function of LARP4 has not been fully elucidated, LARP4 is known to bind poly(A) binding protein PABPC1, an interaction that works to stabilize mRNA [24]. Interestingly, PABPC1 was also identified in complex with PKA–Cα, along with additional RNA-binding proteins (Tables S1 and S2). Although none of these proteins is a known PKA substrate, their ability to complex with PKA-C suggests that PKA may play a role in the regulation of RNA-binding proteins. Importantly, LARP4 has also been implicated as a negative regulator of cancer cell migration and invasion in prostate and breast cancers [32], a process that is more directly relevant to what is known of PKA function.

Several known PKA substrates were enriched in complex with PKA–Cα when active Fyn was present versus in expression of PKA–Cα alone (Figure 1B and Figure 2A), supporting the hypothesis that Fyn-induced phosphorylation of PKA–Cα may enhance PKA activity. In the identification of PKA–Cα interactors induced to associate by Fyn activity, Annexin-A1 (ANXA1) was the most highly enriched binding partner in the in Fyn-active condition (Figure 4A). ANXA1 T216 is a direct substrate of PKA [49], although the consequences of this phosphorylation event remain unexplored. Phosphorylation sites on three PKA regulatory subunits and one AKAP were identified in complex with PKA–Cα (Figure 5C, Table S3, Figure S2). Strikingly, all phosphorylation sites identified were significantly enriched in conditions where wild-type Fyn was co-expressed. PKA-RIα S83 is a known substrate of CDK2/Cyclin E and modification of this site is thought to facilitate dissociation of RIα from the replication factor C complex in the G1/S transition [50]. Phosphorylation of this site and the homologous site of the RIβ isoform, situated within the R subunit linker region, has been hypothesized to serve a regulatory role in release of the catalytic subunits [51]. The increase in phosphorylation of these potential regulatory sites observed in Fyn-active conditions, coupled with the ability of Fyn kinase activity to activate PKA, further supports the potential involvement of these modified sites in a regulatory mechanism, and one that is enhanced by Fyn activity.

## 5. Conclusions

In summary, this study presents Fyn as a novel regulator of PKA–Cα protein–protein interactions. While certain Fyn-enhanced PKA–Cα interactors increased in binding in the presence of active Fyn, the majority of the interactors identified in the quantitative mass spectrometry analyses were Fyn activity-independent. The Fyn-modulated binding of PKA–Cα to three known AKAPs (PDE4DIP, AKAP9, and CDK5RAP2) was characterized biochemically, and the PKA–Cα/AKAP interaction was found to be altered by Fyn catalytic activity and expression levels. In addition, a novel binding partner, LARP4, was shown to bind PKA–Cα in the absence of Fyn when co-expressed in HEK cells. These results contribute to the characterization of a newly identified reciprocal regulatory interaction between the Src family kinase Fyn and PKA.

## Supplementary Materials

The following are available online at http://www.mdpi.com/2227-7382/6/4/37/s1: Table S1: Classification of PKA–Cα binding partners in *PKACα-YFP* and *Fyn WT* (light) or *PKACα-YFP* and *Empty Vector* (heavy) SILAC experiment. Table S2: Classification of PKA–Cα binding partners in *PKACα-YFP* and *Fyn WT* (light) or *PKACα-YFP* and *Fyn KD* (heavy) SILAC experiment. Table S3: Phosphopeptides identified in complex with PKA–Cα. Figure S1: Fyn expression in *PKACα-YFP + Fyn WT* (light) or *PKACα-YFP + Fyn KD* (heavy) SILAC experiment. Figure S2: Fragmentation spectra of phosphorylation sites on proteins in complex with PKA–Cα.
