# Supplementary material for: Fyn Regulates Binding Partners of Cyclic-AMP Dependent Protein Kinase A
Source: Proteomes. 2018 Sep 29;6(4):37. doi: 10.3390/proteomes6040037 (PMC6313912; doi:10.3390/proteomes6040037)
Supplement: Supplementary file 1 [file proteomes-06-00037-s001.pdf]

Supplementary Tables and Figures

Table S1. Classification of PKA-Cα binding partners in *PKACα-YFP* and *Fyn WT* (light) or *PKACα-YFP* and *Empty Vector* (heavy) SILAC experiment

Table S2. Classification of PKA-Cα binding partners in *PKACα-YFP* and *Fyn WT* (light) or *PKACα-YFP* and *Fyn KD* (heavy) SILAC experiment

Table S3. Phosphopeptides identified in complex with PKA-Cα

Figure S1. Fyn expression in *PKACα-YFP* and *Fyn WT* (light) or *PKACα-YFP* and *Fyn KD* (heavy) SILAC experiment.

Figure S2. Fragmentation spectra of phosphorylation sites on proteins in complex with PKA-Cα.

Table S1. Classification of PKA-Cα binding partners in *PKACα-YFP + Fyn WT* (light) or *PKACα-YFP + Empty Vector* (heavy) SILAC experiment.

UNIPROT ACCESSION	GENE SYMBOL	PROTEIN NAME	SPECTRAL COUNT	FDR	LOG_2(H:L)
**NOVEL PKA BINDING PARTNERS**					
**Q53H12**	AGK	Acylglycerol kinase, mitochondrial	5	5.1E-03	-1.005
**O95831**	AIFM1	Apoptosis-inducing factor 1, mitochondrial	7	9.2E-03	-0.714
**Q9NVI7**	ATAD3A	ATPase family AAA domain-containing protein 3A	6	9.4E-05	-1.467
**Q5T9A4**	ATAD3B	ATPase family AAA domain-containing protein 3B	9	9.4E-05	-1.063
**P06576**	ATP5B	ATP synthase subunit beta, mitochondrial	7	5.4E-03	-0.790
**P36542**	ATP5C1	ATP synthase subunit gamma, mitochondrial	5	5.8E-01	-0.150
**P78371**	CCT2	T-complex protein 1 subunit beta	3	8.6E-02	-0.706
**P50991**	CCT4	T-complex protein 1 subunit delta	3	1.5E-02	-1.164
**P48643**	CCT5	T-complex protein 1 subunit epsilon	3	3.3E-03	-1.624
**P50990**	CCT8	T-complex protein 1 subunit theta	8	1.1E-04	-1.141
**Q5SW79**	CEP170	Centrosomal protein of 170 kDa	12	6.3E-09	-1.613
**P31689**	DNAJA1	DnaJ homolog subfamily A member 1	3	1.5E-01	-0.566
**O75190**	DNAJB6	DnaJ homolog subfamily B member 6	4	2.2E-03	-1.355
**O60762**	DPM1	Dolichol-phosphate mannosyltransferase subunit 1	5	1.2E-02	-0.848
**P55010**	EIF5	Eukaryotic transcription initiation factor 5	3	8.0E-09	1.243
**Q15717**	ELAVL1	ELAV-like protein 1	6	2.5E-02	-0.646
**P50402**	EMD	Emerin	6	2.4E-04	-1.318
**P35637**	FUS	RNA-binding protein FUS	4	9.0E-03	-1.052
**P06241**	FYN	Tyrosine-protein kinase Fyn	7	1.4E-09	-3.690
**P09488**	GSTM1	Glutathione S-transferase Mu 1	3	2.1E-03	-1.781
**P28161**	GSTM2	Glutathione S-transferase Mu 2	5	6.5E-04	-1.363
**Q16891**	IMMT	MICOS complex subunit MIC60	12	3.9E-10	-1.889
**Q6PKG0**	LARP1	La-related protein 1	3	8.0E-03	0.691
**Q71RC2**	LARP4	La-related protein 4	9	4.4E-09	0.853
**P20700**	LMNB1	Lamin-B1	3	2.5E-02	-1.032
**Q9BQG0**	MYBBP1A	Myb-binding protein 1A	3	3.2E-01	-0.370
**P35580**	MYH10	Myosin-10	7	8.3E-02	-0.430
**P35579**	MYH9	Myosin-9	9	6.3E-04	-0.894
**O95613**	PCNT	Pericentrin	4	6.8E-03	-1.117
**O43175**	PHGDH	D-3-phosphoglycerate dehydrogenase	12	2.1E-03	-0.632
**P61247**	RPS3A	40S ribosomal protein S3a	7	5.6E-01	-0.135
**P62753**	RPS6	40S ribosomal protein S6	9	8.5E-01	-0.041
**Q3ZCQ8**	TIMM50	Mitochondrial import inner membrane translocase subunit TIM50	5	3.0E-02	-0.686
**Q13748**	TUBA3C	Tubulin alpha-3C/D chain	4	2.8E-01	-0.344
**A6NHL2**	TUBAL3	Tubulin alpha chain-like 3	7	1.1E-02	-0.686
**Q13885**	TUBB2A	Tubulin beta-2A chain	8	1.0E-01	-0.370

**P08670**	VIM	Vimentin	35	5.4E-19	-1.459
**KNOWN PKA BINDING PARTNERS**					
**Q92667**	AKAP1	A-kinase anchor protein 1, mitochondrial	6	8.9E-01	-0.034
**Q9UKA4**	AKAP11	A-kinase anchor protein 11	71	5.1E-05	0.282
**Q12802**	AKAP13	A-kinase anchor protein 13	4	6.5E-04	0.754
**Q9Y2D5**	AKAP2	A-kinase anchor protein 2	13	3.6E-01	0.139
**P24588**	AKAP5	A-kinase anchor protein 5	10	7.5E-06	-1.208
**Q99996**	AKAP9	A-kinase anchor protein 9	607	2.0E-176	-1.427
**Q16543**	CDC37	Hsp90 co-chaperone Cdc37	39	1.3E-13	-0.952
**Q96SN8**	CDK5RAP2	CDK5 regulatory subunit-associated protein 2	52	1.5E-28	-1.579
**P07900**	HSP90AA1	Heat shock protein HSP 90-alpha	110	6.1E-28	-0.924
**P08238**	HSP90AB1	Heat shock protein HSP 90-beta	57	1.4E-18	-0.951
**P11137**	MAP2	Microtubule-associated protein 2	7	1.6E-02	0.461
**Q15691**	MAPRE1	Microtubule-associated protein RP/EB family member 1	13	1.7E-08	-1.416
**Q5VU43**	PDE4DIP	Myomegalin	164	6.9E-58	-1.287
**P22694**	PRKACB	cAMP-dependent protein kinase catalytic subunit beta	31	1.5E-01	-0.161
**P10644**	PRKAR1A	cAMP-dependent protein kinase type I-alpha regulatory subunit	148	3.7E-11	0.522
**P31321**	PRKAR1B	cAMP-dependent protein kinase type I-beta regulatory subunit	17	1.7E-08	0.643
**P13861**	PRKAR2A	cAMP-dependent protein kinase type II-alpha regulatory subunit	282	1.5E-19	-0.467
**P31323**	PRKAR2B	cAMP-dependent protein kinase type II-beta regulatory subunit	61	9.0E-19	-0.921
**KNOWN PKA SUBSTRATES**					
**Q12802**	AKAP13	A-kinase anchor protein 13	4	6.5E-04	0.754
**Q99996**	AKAP9	A-kinase anchor protein 9	607	2.0E-176	-1.427
**O60762**	DPM1	Dolichol-phosphate mannosyltransferase subunit 1	5	1.2E-02	-0.848
**P50402**	EMD	Emerin	6	2.4E-04	-1.318
**P06241**	FYN	Tyrosine-protein kinase Fyn	7	1.4E-09	-3.690
**P07900**	HSP90AA1	Heat shock protein HSP 90-alpha	110	6.1E-28	-0.924
**P08238**	HSP90AB1	Heat shock protein HSP 90-beta	57	1.4E-18	-0.951
**Q16891**	IMMT	MICOS complex subunit MIC60	12	3.9E-10	-1.889
**P11137**	MAP2	Microtubule-associated protein 2	7	1.6E-02	0.461
**P13861**	PRKAR2A	cAMP-dependent protein kinase type II-alpha regulatory subunit	282	1.5E-19	-0.467
**P31323**	PRKAR2B	cAMP-dependent protein kinase type II-beta regulatory subunit	61	9.0E-19	-0.921
**P08670**	VIM	Vimentin	35	5.4E-19	-1.459

Table S2. Classification of PKA-Cα binding partners in *PKACα-YFP + Fyn WT* (light) or *PKACα-YFP + Fyn KD* (heavy) SILAC experiment.

UNIPROT ACCESSION	GENE SYMBOL	PROTEIN NAME	SPECTRAL COUNT	FDR	$LOG_2(H:L)$
**NOVEL PKA BINDING PARTNERS**					
**P23526**	AHCY	Adenosylhomocysteinase	4	4.8E-44	0.772
**P04075**	ALDOA	Fructose-bisphosphate aldolase A	3	4.6E-22	0.579
**P04083**	ANXA1	Annexin A1	12	2.3E-111	-3.043
**P27708**	CAD	CAD protein	3	1.6E-10	0.395
**Q86VP6**	CAND1	Cullin-associated NEDD8-dissociated protein 1	4	2.4E-75	1.576
**P40227**	CCT6A	T-complex protein 1 subunit zeta	3	6.1E-38	0.772
**P55060**	CSE1L	Exportin-2	4	3.9E-63	0.945
**Q92841**	DDX17	Probable ATP-dependent RNA helicase DDX17	4	1.4E-46	0.773
**Q14204**	DYNC1H1	Cytoplasmic dynein 1 heavy chain 1	10	2.3E-61	0.659
**P60842**	EIF4A1	Eukaryotic initiation factor 4A-I	6	8.0E-59	0.771
**Q92616**	GCN1L1	eIF-2-alpha kinase activator GCN1	9	1.7E-50	0.880
**O60812**	HNRNPCL1	Heterogeneous nuclear ribonucleoprotein C-like 1	3	5.0E-05	0.254
**O00425**	IGF2BP3	Insulin-like growth factor 2 mRNA-binding protein 3	3	8.9E-06	0.277
**O00410**	IPO5	Importin-5	4	1.4E-35	0.665
**Q7Z794**	KRT77	Keratin, type II cytoskeletal 1b	5	1.2E-55	-1.862
**Q659C4**	LARP1B	La-related protein 1B	5	8.1E-03	-0.146
**Q71RC2**	LARP4	La-related protein 4	5	6.7E-24	0.517
**Q9BQG0**	MYBBP1A	Myb-binding protein 1A	4	2.1E-37	0.693
**P11940**	PABPC1	Polyadenylate-binding protein 1	33	1.1E-39	0.296
**Q13310**	PABPC4	Polyadenylate-binding protein 4	9	1.0E-09	0.227
**P78527**	PRKDC	DNA-dependent protein kinase catalytic subunit	60	3.9E-117	0.583
**P62269**	RPS18	40S ribosomal protein S18	4	3.9E-21	0.499
**O75746**	SLC25A12	Calcium-binding mitochondrial carrier protein Aralar1	9	3.6E-116	1.098
**Q9UJS0**	SLC25A13	Calcium-binding mitochondrial carrier protein Aralar2	7	9.9E-72	0.912
**Q12931**	TRAP1	Heat shock protein 75 kDa, mitochondrial	3	3.3E-08	0.340
**Q13509**	TUBB3	Tubulin beta-3 chain	4	1.5E-23	0.529
**Q9HAV4**	XPO5	Exportin-5	3	8.5E-37	0.759
**KNOWN PKA BINDING PARTNERS**					
**Q92667**	AKAP1	A-kinase anchor protein 1, mitochondrial	3	7.1E-05	0.250
**Q9UKA4**	AKAP11	A-kinase anchor protein 11	38	3.2E-12	0.152
**Q12802**	AKAP13	A-kinase anchor protein 13	7	2.6E-16	0.342
**P24588**	AKAP5	A-kinase anchor protein 5	4	6.6E-96	1.209
**Q99996**	AKAP9	A-kinase anchor protein 9	354	2.1E-14	0.099
**Q16543**	CDC37	Hsp90 co-chaperone Cdc37	18	1.6E-08	-0.177
**Q96SN8**	CDK5RAP2	CDK5 regulatory subunit-associated protein 2	41	6.6E-02	0.045
**Q5VU43**	PDE4DIP	Myomegalin	71	6.6E-06	0.075

**P10644**	PRKAR1A	cAMP-dependent protein kinase type I-alpha regulatory subunit	64	6.0E-01	-0.009
**P31321**	PRKAR1B	cAMP-dependent protein kinase type I-beta regulatory subunit	10	3.2E-11	0.377
**P13861**	PRKAR2A	cAMP-dependent protein kinase type II-alpha regulatory subunit	173	7.9E-68	0.252
**P31323**	PRKAR2B	cAMP-dependent protein kinase type II-beta regulatory subunit	23	4.7E-16	0.201
**O14980**	XPO1	Exportin-1	13	4.1E-63	0.615
**KNOWN PKA SUBSTRATES**					
**Q12802**	AKAP13	A-kinase anchor protein 13	7	2.6E-16	0.342
**Q99996**	AKAP9	A-kinase anchor protein 9	354	2.1E-14	0.099
**P04075**	ALDOA	Fructose-bisphosphate aldolase A	3	4.6E-22	0.579
**P04083**	ANXA1	Annexin A1	12	2.3E-111	-3.043
**P27708**	CAD	CAD protein	3	1.6E-10	0.395
**P13861**	PRKAR2A	cAMP-dependent protein kinase type II-alpha regulatory subunit	173	7.9E-68	0.252
**P31323**	PRKAR2B	cAMP-dependent protein kinase type II-beta regulatory subunit	23	4.7E-16	0.201

Table S3. Phosphopeptides identified in complex with PKA-Cα. Experimental conditions each phosphopeptide was identified in is indicated, along with the PKA-Cα-normalized H:L ratio for that peptide. Amino acid modifications (phosphorylation = *, heavy isotopes of carbon and nitrogen = #) are indicated after the modified amino acid in the given peptide. Annotated spectra are included in Figure S2. H:L ratios of all phosphopeptides identified were statistically lower ($p < 0.05$) from that of the average H:L for each individual protein. Spectral counts (*) refer to the sum of spectra assigned to peptides housing the indicated phosphorylation site, including heavy and light peptides as well as alternative cleavages.

Light SILAC Condition	Heavy SILAC Condition	Gene Name	Log_2(H:L)	Peptide	Site	Spectrum (Figure S2)	Spectral Count (pY)*
YFP-PKACA + Fyn WT	*YFP-PKACA + EV*	**AKAP11**	-0.431	SVS*PTFLNPSDENLK	**S1242**	A	2
YFP-PKACA + Fyn WT	*YFP-PKACA + EV*	**PRKAR1A**	-0.731	TDSREDEIS*PPPPNPVVK	**S83**	B	2
YFP-PKACA + Fyn WT	*YFP-PKACA + Fyn KD*	**PRKAR1A**	-2.470	EDEIS*PPPPNPVVK#	**S83**	C	4
YFP-PKACA + Fyn WT	*YFP-PKACA + Fyn KD*	**PRKAR1B**	-2.211	SNSQSDSHDEEVS*PTPPNPVVK	**S83**	D	1
YFP-PKACA + Fyn WT	*YFP-PKACA + Fyn KD*	**PRKAR2A**	-2.861	VADAKGDS*ESEEDEDLEVPVPSR	**S78**	E	2
YFP-PKACA + Fyn WT	*YFP-PKACA + Fyn KD*	**PRKAR2A**	-3.041	VADAKGDS*ES*EEDEDLEVPVPSR	**S78, S80**	F	1

Figure S1. Fyn expression in *PKACα-YFP + Fyn WT* (light) or *PKACα-YFP + Fyn KD* (heavy) SILAC experiment. Western blots of whole cell lysates from this SILAC experiment demonstrate even levels of PKA-Cα (α-GFP) and Fyn WT/KD (α-Fyn). The α-pY blot confirms Fyn kinase activity in the conditions where Fyn WT is expressed. A strong signal is also observed at 75 kDa in the condition where Fyn WT and PKA-Cα were co-expressed.

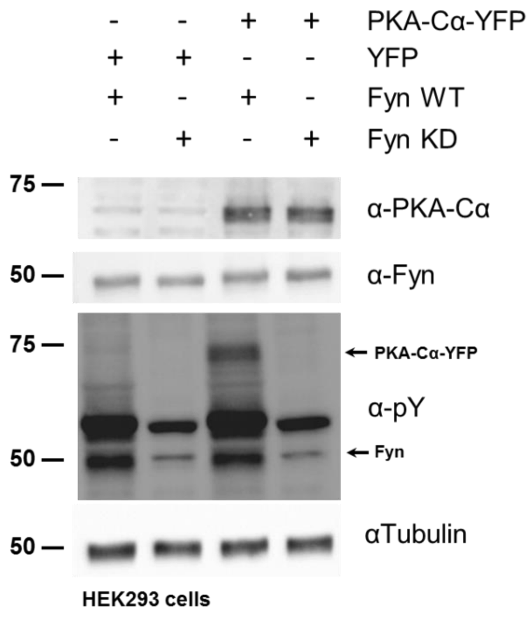

Figure S2. Fragmentation spectra of phosphorylation sites on proteins in complex with PKA-Cα. Spectra we acquired in a linear ion trap mass analyzer. Peptide charge state is indicated. Amino acid modifications (phosphorylation = *, heavy isotopes of carbon and nitrogen = #) are indicated after the modified amino acid in the given peptide.

A

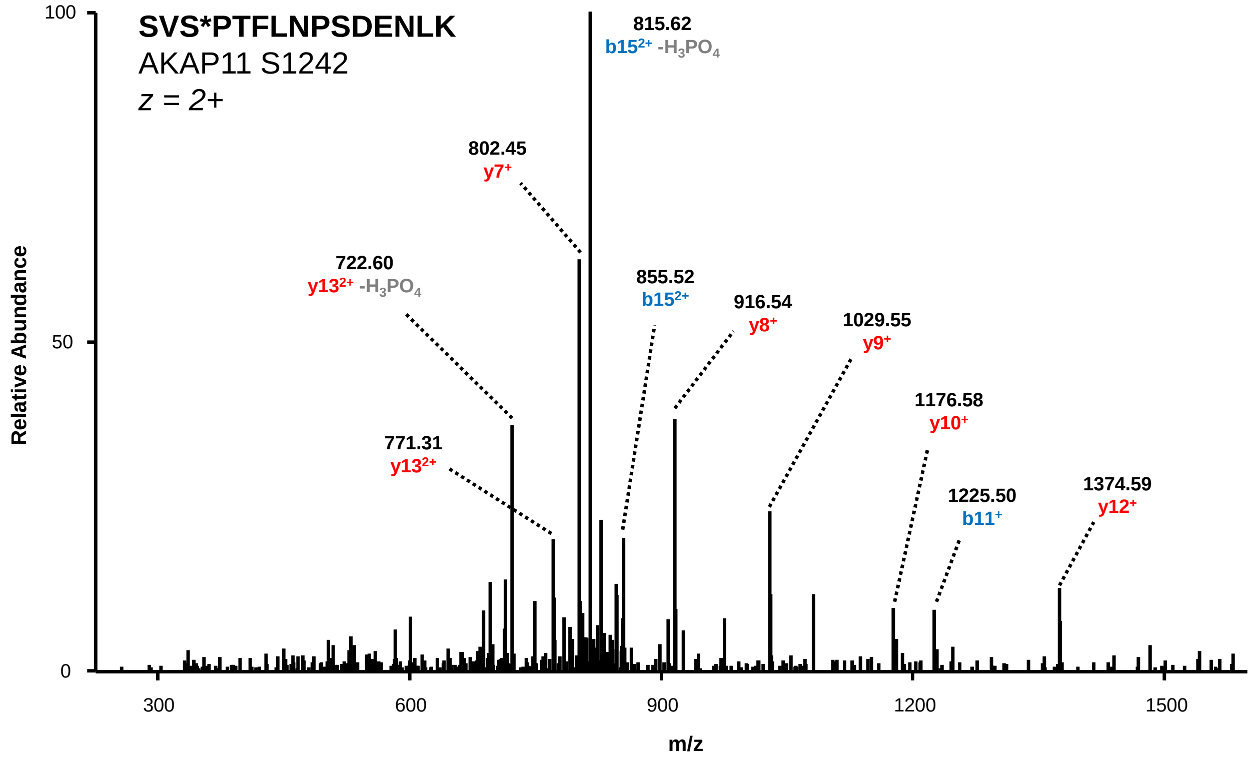

B

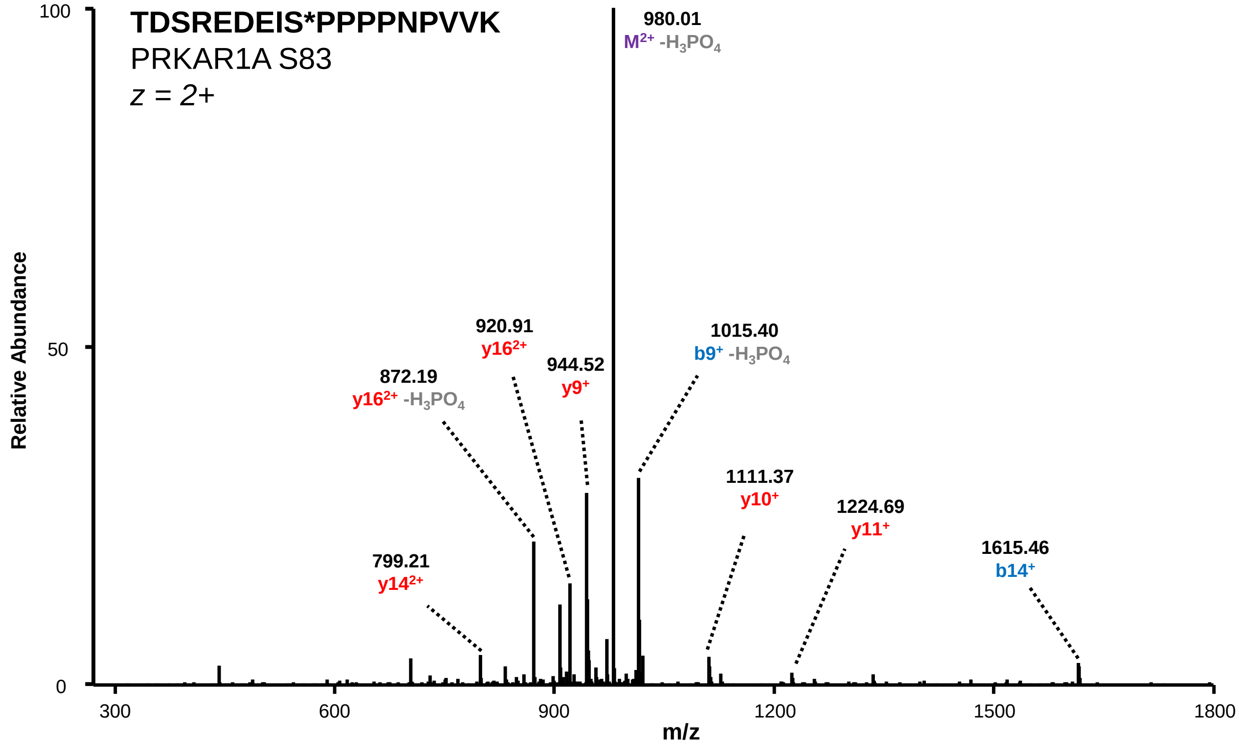

C

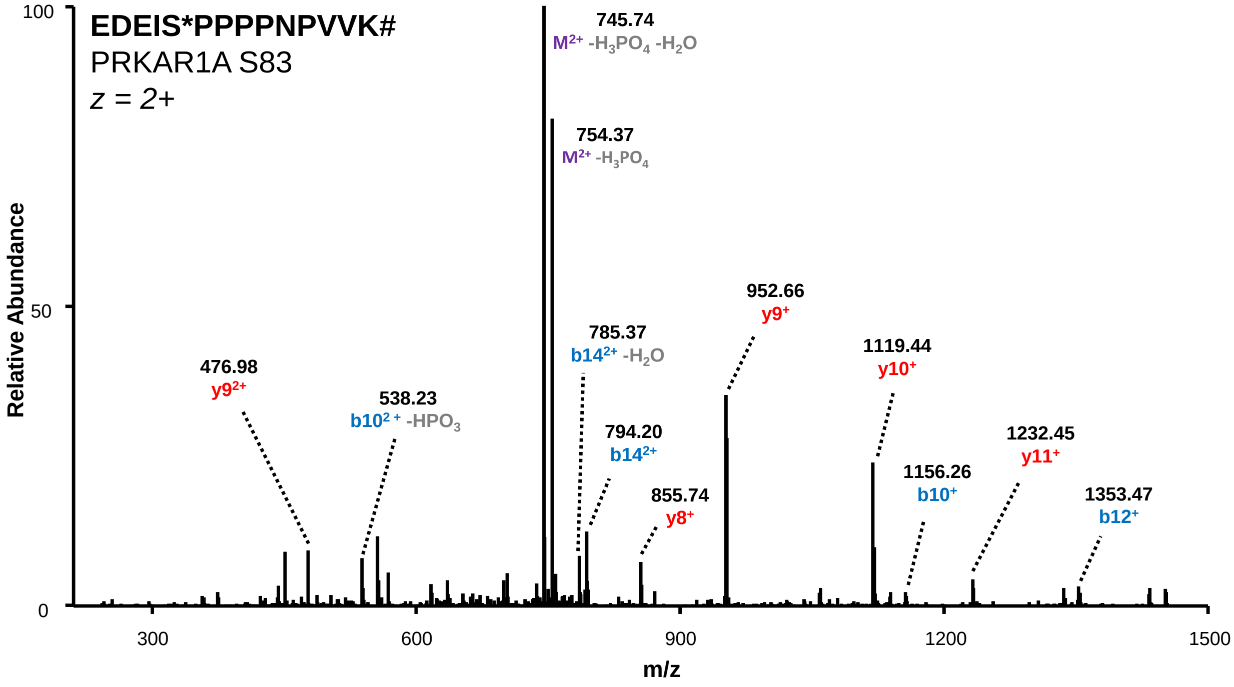
EDEIS*PPPPNPVVK#
PRKAR1A S83
z = 2+
745.74
M2+ -H3PO4 -H2O
754.37
M2+ -H3PO4
952.66
y9+
785.37
b142+ -H2O
1119.44
y10+
476.98
y92+
538.23
b102 + -HPO3
794.20
b142+
1232.45
y11+
1156.26
b10+
855.74
y8+
1353.47
b12+
Relative Abundance
100
50
0
300
600
900
1200
1500
m/z

D

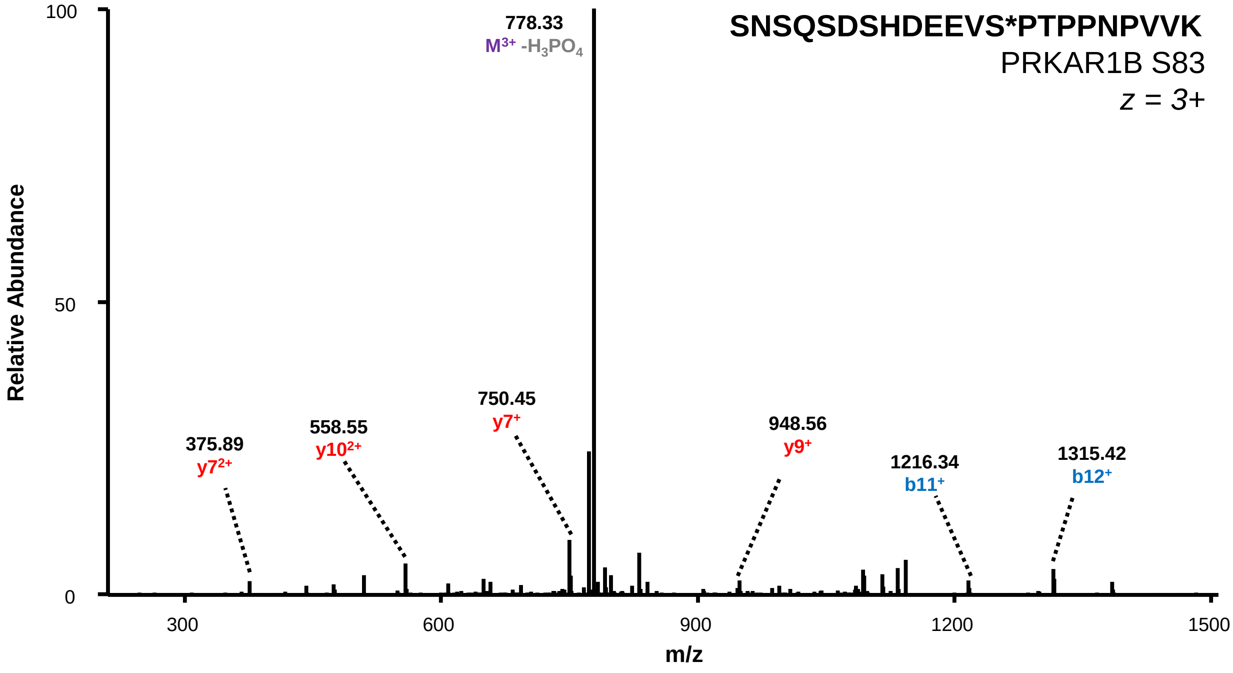
778.33
M3+ -H3PO4
SNSQSDSHDEEVS*PTPPNPVVK
PRKAR1B S83
z = 3+
750.45
y7+
558.55
y102+
375.89
y72+
948.56
y9+
1216.34
b11+
1315.42
b12+
Relative Abundance
100
50
0
300
600
900
1200
1500
m/z

E

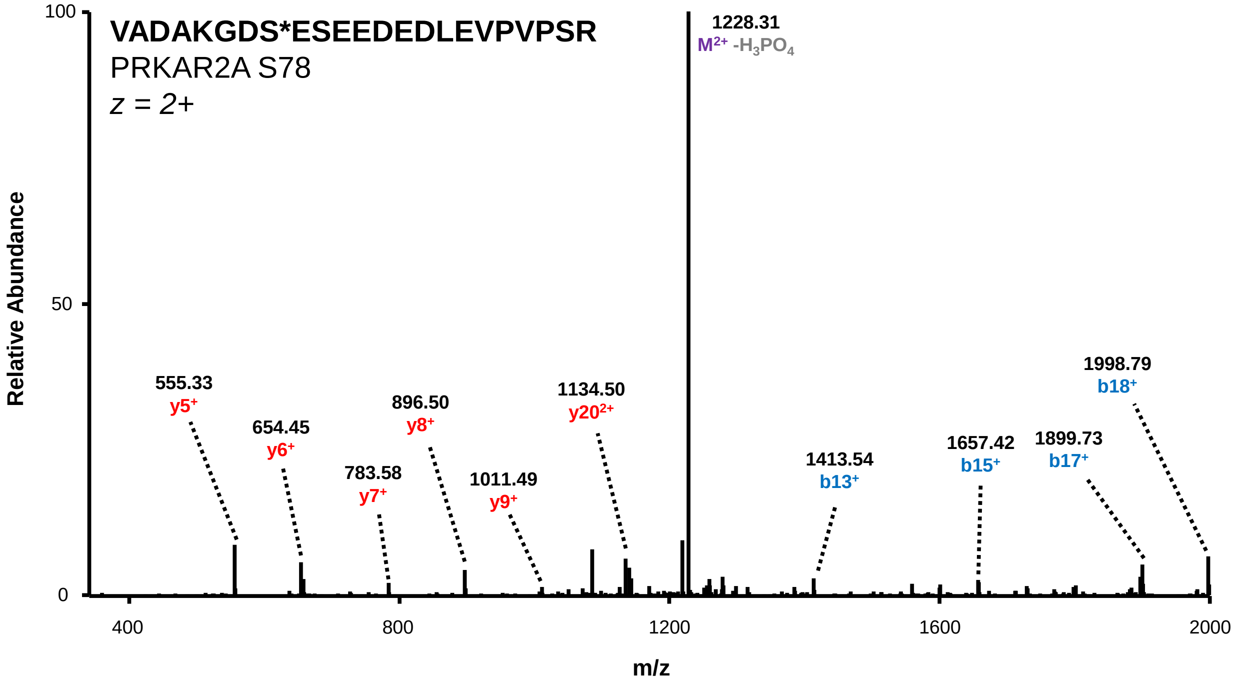

VADAKGDS*ESEEDEDLEVPVPSR
PRKAR2A S78
z = 2+
Relative Abundance
100
50
0
1228.31
M2+ -H3PO4
555.33
y5+
654.45
y6+
783.58
y7+
896.50
y8+
1011.49
y9+
1134.50
y20 2+
1413.54
b13+
1657.42
b15+
1899.73
b17+
1998.79
b18+
400
800
1200
1600
2000
m/z

F

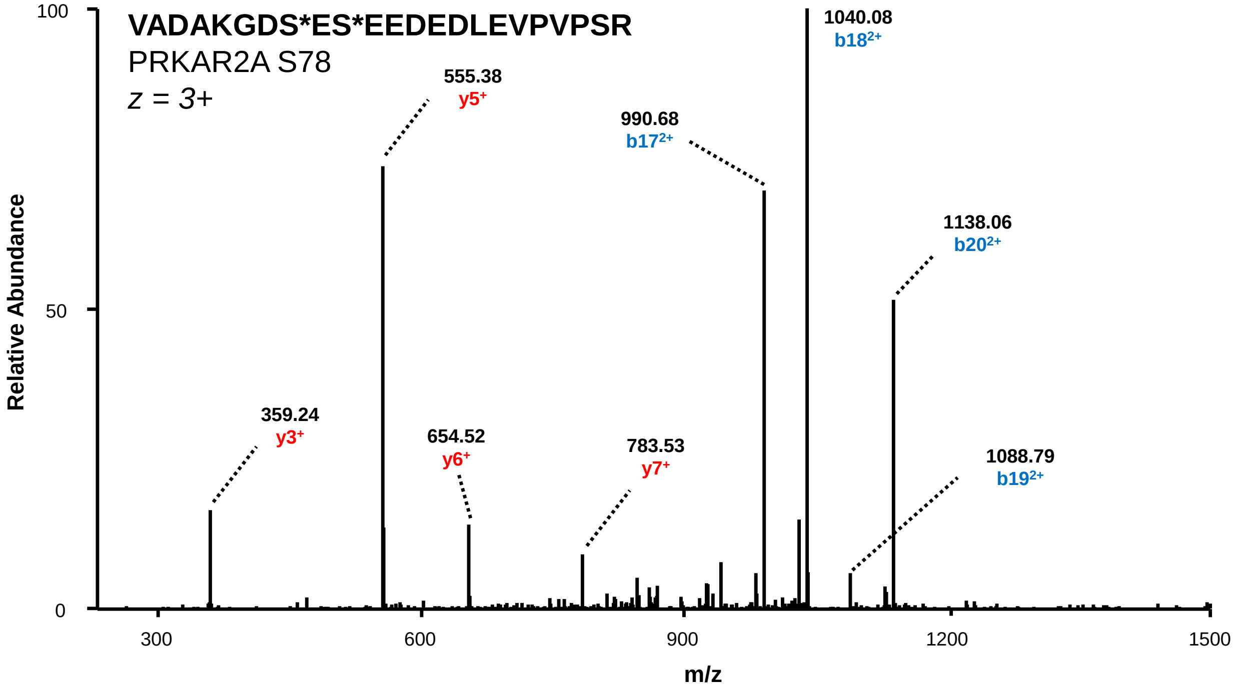

VADAKGDS*ES*EEDEDLEVPVPSR
PRKAR2A S78
z = 3+
Relative Abundance
100
50
0
1040.08
b18 2+
555.38
y5+
990.68
b17 2+
1138.06
b20 2+
359.24
y3+
654.52
y6+
783.53
y7+
1088.79
b19 2+
300
600
900
1200
1500
m/z
